# Comparative evaluation of major cropping patterns in coastal saline-alkali lands: Economic, ecological, and social benefits

**DOI:** 10.1371/journal.pone.0353467

**Published:** 2026-07-21

**Authors:** Hongliang Ma, Dongxu Yang, Maoxing Song, Shangqing Zhang, Chen Wang, Mengying Yang, Tong Li, Zhengyu Yin, Yunting Bian, Cheng Peng, Mochu Wang, Peng Wu, Xiaoxue Zhao, Guomin Yuan, Qing Lu, Zhihui Wu

**Affiliations:** 1 Tangshan Academy of Agricultural Sciences, Tangshan, China; 2 Chinese Academy of Agricultural Sciences, Beijing, China; 3 College of Animal Science and Technology, Hebei Agricultural University, Baoding, Hebei, China; CSSRI: Central Soil Salinity Research Institute, INDIA

## Abstract

Soil salinization constrains agricultural productivity in coastal regions. A two-year field experiment in the coastal saline-alkali farmland of Tangshan, China evaluated three locally adapted cropping patterns: fresh edible pea-rice rotation, rice-crab co-culture, and wheat-rice rotation, with conventional monocropping systems as controls. The fresh edible pea-rice rotation increased net income by 94.78% compared with rice monoculture. The rice-crab co-culture generated the highest economic return, with net income ranging from 24,000–53,400 CNY ha ^−^ ^1^. The wheat-rice rotation increased net income by 49.8% compared with wheat-maize rotation and by 180.64% compared with rice monoculture. In addition, the cropping patterns showed trends of reduced soil salinity (by 0.13%–0.20% in total soluble salt content) and increased soil organic matter (by 0.20–0.30 percentage points) relative to controls, significantly reduced pesticide inputs by 20%–50% (P < 0.05), and exhibited nitrogen and phosphorus loss reduction rates of approximately 15%–25%. These results demonstrate that optimized cropping patterns can simultaneously enhance economic performance and ecological sustainability in coastal saline-alkali agriculture, with soil quality improvements showing promising trends over the two-year study period.

## 1. Introduction

Soil salinization is one of the major challenges facing global agricultural development, directly threatening food security and agricultural sustainability. According to the Global Map of Salt-Affected Soils (GSASmap) released by the FAO in 2021, the map covers 85% of the global land surface. The data shows that over 833 million hectares (approximately 8.7% of the Earth's land area) are affected by salinization.[1,2]. In China, coastal saline-alkali land represents a distinct land resource type mainly distributed in the eastern coastal region, with Tangshan being a typical example. In 2023, based on high-resolution satellite imagery interpretation and field surveys, Hebei Province identified 380,167 hectares of saline-alkali arable land, of which 50,687 hectares were located in the Tangshan area, indicating substantial development potential [3] (Table 1).

**Table 1:** Statistics on classification of water-soluble salt in plow layer soil and area of saline-alkali cultivated land.

County/District	Agricultural Land Area (ha)	Grade 1	Grade 2	Grade 3	Grade 4	Grade 5
		(≥0.6%)	(0.6%−0.4%)	(0.4%−0.2%)	(0.2%−0.1%)	(≤0.1%)
Haixing County	33,950	9,298.54	3,039.64	4,157.15	14,244.38	3,210.30
Huanghua City	110,631	18,824.33	8,475.89	31,778.89	38,256.32	13,295.57
Tanghai County	43,313.80	8,634.52	10,384.13	12,801.53	7,358.50	4,134.70
Hangu District	12,411.80	–	–	3,403.01	6,023.19	2,985.60
Lutai District	10,184.60	–	–	–	7,016.20	3,168.40
Fengnan District	80,251.50	2,414.95	6,653.07	8,839.90	29,399.50	32,944.44
Yanshan County	60,510.60	–	–	2,085.46	28,169.34	30,255.30
Luannan County	100,746	9,554.83	827.26	3,340.84	16,134.28	70,888.79
Laoting County	88,552.40	211.2	1,287.82	15,380.71	11,282.23	60,390.92
Changli County	91,066.40	–	–	98	1,060.13	89,908.27
Funing County	118,592	–	–	–	618.2	117,973.80
Qinhuangdao City	23,165.10	–	–	–	212.3	22,952.80
Total	773,375.20	48,938.37	30,667.81	81,885.49	159,774.60	452,108.89
Percentage of Total (%)	–	6.33	3.97	10.59	20.66	58.46

The Food and Agriculture Organization (FAO) has established multiple networks and working groups dedicated to addressing soil salinization, including the International Network of Salt-affected Soils (INSAS), the Global Framework on Water Scarcity in Agriculture (WASAG), the Saline Agriculture Working Group (SAWG), and the Global Alliance for Climate-Smart Agriculture (GACSA) [4]. These platforms serve as key mechanisms for sharing knowledge and fostering collaborative efforts in the development and implementation of innovative saline-alkali land management strategies. Complementing these initiatives, the United States Department of Agriculture (USDA) operates the internationally renowned Salinity Laboratory under its Agricultural Water Efficiency and Salinity Research Unit [5]. This institution focuses on advancing methodologies and management practices to assess, predict, and regulate the transport of water, salts, pesticides, and microorganisms within the root-zone soils of arid regions.

Coastal saline-alkali lands represent important reserve agricultural resources in China, particularly in the Bohai Bay region. However, high soil salinity, poor soil structure, and nutrient imbalance severely constrain crop productivity and ecological stability. In recent years, increasing attention has been paid to the development of sustainable cropping systems for saline-alkali agriculture, aiming to simultaneously improve soil quality, agricultural productivity, and farmers’ income. Among various management approaches, crop rotation systems and integrated co-culture systems have shown considerable potential due to their ecological and economic advantages.

Research on the reclamation and utilization of saline-alkali land has been conducted for many years, covering physical, chemical, and biological amelioration techniques, as well as optimization of planting systems such as rotation, intercropping, and relay intercropping. For example, biotechnological approaches, such as breeding salt-tolerant varieties and applying biofertilizers, have been shown to improve crop yields and soil quality [6]. Other studies have used ecological engineering methods, including constructing drainage systems and implementing bioremediation, to improve ecosystem conditions in saline-alkali lands [7,8].

As the Chinese government has placed increasing emphasis on the comprehensive utilization of saline-alkali land [9], various regions have conducted extensive research and practical exploration. Regarding cropping models for coastal saline-alkali land, different areas have developed distinctive systems based on local natural conditions and resource endowments. For instance, Panjin in Liaoning has developed a rice-crab co-culture model that achieves both economic and ecological benefits [10,11] through the mutualistic relationship between rice and river crabs. In parts of the Yangtze River Basin, wheat-rice rotation has become an important approach for enhancing paddy productivity and improving soil properties.

Although several cropping systems and crop rotations have been widely applied in saline-alkali regions of China [12–14], most previous studies have focused on individual systems or site-specific demonstrations. Systematic comparisons of multiple cropping patterns under the same coastal saline-alkali conditions remain limited. In particular, quantitative evaluations integrating economic performance, soil improvement, and ecological indicators across different cropping systems are still scarce.

**Fig 1:**
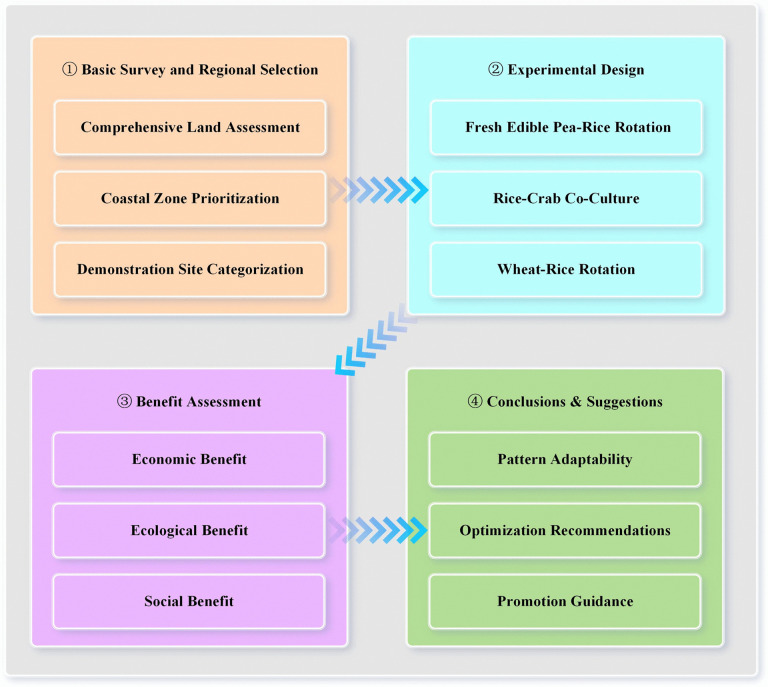
Experimental design for three major planting patterns in tangshan coastal saline-alkali lands.

This study provides a comparative assessment of three representative planting patterns under identical environmental and management conditions in Tangshan coastal saline-alkali farmland. By integrating economic returns, soil physicochemical changes, and ecological indicators, this research aims to clarify the relative advantages and ecological-economic trade-offs among rotation systems and co-culture systems, thereby contributing new empirical evidence for optimizing cropping strategies in coastal saline-alkali agriculture. This study was designed to address three key scientific questions: (i) how different planting patterns regulate the coupled responses of soil salinity reduction, soil organic matter accumulation, and economic returns under moderate to strongly saline-alkali stress (total salt content 0.1%–0.5%, EC₁:₅ 2–4 dS m ^−^ ^1^); (ii) how rotation systems and co-culture systems differ in terms of ecological-economic efficiency and input-output balance in saline-alkali farmlands; and (iii) what short-term (two-year) quantitative response ranges can be observed for soil improvement indicators under different planting patterns(Fig 1).

## 2. Materials and methods

### 2.1. Site characteristics

The study area is located in the coastal saline-alkali region of Tangshan, Hebei Province, China (39°20′–39°50′N, 118°20′–118°50′E). The region has a warm temperate monsoon climate with an average annual temperature of approximately 11.2 °C, an average annual precipitation of about 610 mm (mainly concentrated from June to September), and an average annual evaporation of 1,700–1,900 mm, typical of a semi-humid coastal saline-alkali zone.

It should be noted that soil salinity classification criteria vary across regions and analytical methods [15,16]. To ensure international comparability and consistency with widely accepted frameworks, this study adopts the USDA salinity hazard classification referenced by Pokhrel et al. [15], which defines salinity categories based on electrical conductivity of saturated paste extract (ECe): nonsaline (ECe < 2 dS m ^−^ ^1^), very slightly saline (ECe 2–4 dS m ^−^ ^1^), and saline (ECe > 4 dS m ^−^ ^1^). For coastal saline soils, ECe values are typically 4–5 times higher than EC₁:₅ values (1:5 soil-to-water extract), owing to the larger dilution factor inherent in fixed-ratio water extracts. Pokhrel et al. demonstrated in Mid-Atlantic coastal soils (Delmarva Peninsula) that EC₁:₅ and ECe exhibit a strong positive linear relationship (r² = 0.77–0.91, P < 0.0001) and proposed that routine agronomic soil tests (e.g., Mehlich-3 extractable Na) can serve as a cost-effective proxy for salinity classification in coastal environments. Applying this framework to the present study, the experimental soil had an EC₁:₅ of 4.25 dS m ^−^ ^1^, which corresponds to an estimated ECe of approximately 17–21 dS m ^−^ ^1^. This value substantially exceeds the ECe > 4 dS m ^−^ ^1^ threshold for saline soils. Therefore, the experimental soil is classified as strongly saline under the USDA system.

### 2.2. Plant and animal materials

The pea materials used in this study were obtained from the edible bean research team of the Tangshan Academy of Agricultural Sciences, including Tangwan 2 and Tangwan 3 (salt-tolerant, high-yield, early-maturing varieties) and Zhongwan 6 and Zhongwan 9 (traditional elite varieties for mildly saline-alkali plots). The wheat materials were provided by the Wheat Research Institute of the Tangshan Academy of Agricultural Sciences, with Xinmai 296 as the main variety. The rice materials were sourced from the Rice Experimental Station of the Tangshan Academy of Agricultural Sciences, including Kenyu 60, Bindao 9, Longjing 59, Tianlongyou 619, Jinyuan 89, Haiyu 7233, Jinyuan U99, and Xiangjing 5. The crab materials were Chinese mitten crabs (Eriocheir sinensis) obtained from the Tangshan Fisheries Station. All materials were used in accordance with local agricultural and aquaculture regulations, and no protected or endangered species were involved in this study.

### 2.3. Experimental design

#### 2.3.1. Experimental sites.

Fresh edible pea-rice rotation: Demonstration site was located at Laoting County Guyhe Breeding Farm, Tangshan, with a demonstration scale of 3.33 ha. Laoting is the largest production and processing base for fresh edible peas in North China, with 5,333.33 ha of saline-alkali paddy fields and ample spring fallow land. For each planting pattern, the experimental area was arranged using a randomized block design. Each treatment consisted of three replicated plots, and each plot was managed as an independent experimental unit to ensure statistical reliability.

Rice-crab co-culture: Experimental base was located at Tangshan Daomanyuan Agricultural Science and Technology Co., Ltd., with a demonstration area of 13.33 ha. The site has suitable soil and water conditions for rice-crab cultivation and complete paddy-field infrastructure.

Wheat-rice rotation: Demonstration bases were located at the Huapu Grain Planting Specialized Cooperative and the Ze’en Rice Planting Specialized Cooperative in Luannan County, with a total demonstration area of 233.33 ha. Luannan County is a key distribution area of coastal saline-alkali land in Tangshan, with representative soil and climate conditions.

#### 2.3.2. Plot design and replication.

For each planting pattern, the experimental area was arranged using a randomized block layout. Individual plots were treated as independent management units, each receiving identical agronomic management within treatments but independent field operations across plots.

Each treatment consisted of three replicated plots. Plot size was 30 m × 40 m (1,200 m²) for the fresh edible pea-rice rotation and rice-crab co-culture systems, and 50 m × 60 m (3,000 m²) for the wheat-rice rotation system. The experimental plots were embedded within larger demonstration fields. To minimize edge effects and cross-interference, a randomized block design was adopted for all experiments. Each treatment contained three replicated plots separated by 1.5 m buffer zones to minimize edge effects and water-fertilizer interference among treatments. For the fresh edible pea-rice rotation and rice-crab co-culture, each plot measured 0.1 ha (30 m × 33 m). For the wheat-rice rotation and its control treatments (wheat-maize rotation and rice monoculture), each plot measured 0.2 ha (40 m × 50 m) to accommodate both seasonal crops within the same plot. Soil samples were collected from five randomly selected points within each plot using a soil auger (0–20 cm depth) and composited into one representative sample per plot at the beginning and end of each growing season. Plant yield was determined by harvesting the entire plot or from fixed-area subplots (25 m²) with three replicates per plot.

In the wheat-rice rotation experiment, two control treatments (wheat-maize rotation and rice monoculture) were included and arranged following the same randomized block design and replication scheme. This design ensured that observed differences among treatments reflected treatment effects rather than field heterogeneity.

#### 2.3.3. Study duration.

The study was conducted from 2022 to 2023, covering two consecutive growing seasons to ensure the reliability and stability of the experimental results.

#### 2.3.4. Data collection procedures.

Yield data: At maturity, the entire plot was harvested to determine the yield of crops (fresh edible peas, rice, wheat) and aquatic products (crabs). For crops, grain moisture content was measured using a moisture analyzer, and yield was converted to the standard moisture content (14.5% for rice and wheat, fresh weight for fresh edible peas). For crabs, the total weight and number per plot were recorded to calculate yield per unit area.

Soil samples were collected from the plow layer (0–20 cm depth) before sowing and after harvest each year. Soil salinity was determined by the weight method, soil organic matter by the potassium dichromate oxidation method, soil bulk density by the ring knife method, and soil porosity by calculation (1 – bulk density/particle density).

Agrochemical input data: The types and amounts of chemical pesticides, fertilizers, and other agrochemicals used in each plot were recorded in detail to calculate the total input per unit area.

Biodiversity data: For the rice-crab co-culture model, aquatic insects, plankton, and soil microorganisms were sampled using standard methods. Aquatic insects and plankton were collected using a plankton net (25 mesh) and identified and counted under a microscope. Soil microorganisms were analyzed for community diversity using high-throughput sequencing.

#### 2.3.5. Soil and yield sampling.

For soil analysis, five subsamples were randomly collected from each plot at a depth of 0–20 cm using an auger and then composited into one representative sample per plot. Soil sampling was conducted before sowing and after harvest each year. For yield determination, all plants within the central harvest area of each plot were collected to avoid border effects. Crop yield was converted to standard moisture content before statistical analysis.

The three cropping systems were selected based on their representativeness and current adoption in coastal saline-alkali agriculture in Tangshan. The fresh edible pea-rice rotation represents an intensive double-cropping system designed to improve land-use efficiency and soil fertility through legume incorporation. The rice-crab co-culture system represents an ecological integrated farming model combining rice cultivation and aquaculture. The wheat-rice rotation represents a grain-oriented annual rotation system widely practiced in moderately to strongly saline coastal farmlands. Conventional monocropping or local conventional rotation systems were used as controls for comparison.

### 2.4. Cultivation and management techniques

#### 2.4.1. Fresh edible pea-rice rotation.

Fresh edible pea cultivation: Sowing was conducted in early March (surface soil temperature stable at 0–5°C) with a seeding rate of 225 kg ha ^−^ ^1^, row spacing of 30 cm, and plant spacing of 5 cm. Basal fertilization was applied before sowing (organic fertilizer combined with nitrogen, phosphorus, and potassium fertilizers), and topdressing was performed once at early flowering. Weed control adopted pre-emergence and post-emergence herbicides in accordance with local agricultural standards, and pest control focused on leaf miners with low-toxicity pesticides (spraying stopped 15 days before harvest). Harvesting was completed in early June when over 70% of pods turned light green.

Rice cultivation: Seedling raising was conducted in late March using factory-style micro-sprinkler dry-bed methods. Transplanting was carried out in mid-June after pea harvest, with a planting density of 3–4 seedlings per hill and row spacing of 16 cm × 30 cm. Basal fertilizer was applied as controlled-release fertilizer, and topdressing was performed at the greening, tillering, and panicle initiation stages. Water management followed the principle of “deep water for seedling protection, shallow water for tillering, mid-season drainage, and alternate wetting and drying during grain filling.” During the experimental period, disease and pest management was performed according to local agronomic practices using low-toxicity pesticides when necessary. Harvesting was conducted in mid-late October when over 95% of grains matured.

#### 2.4.2. Rice-crab co-culture.

Paddy field engineering: An annular trench (2−3 m wide, 0.8−1 m deep, side slope ratio 1:1.5) accounting for 10%−15% of the total paddy area was excavated around the field, with “ + ” or “#” shaped field ditches in the middle. Field bunds were raised to 0.6–0.8 m high, and double-layer escape-prevention screens were installed at inlets and outlets.

Rice cultivation: Seedling raising was conducted in early April using dry-bed methods. Transplanting was carried out from late May to early June with a wide-narrow row pattern (wide row 40 cm, narrow row 20 cm), plant spacing of 16–20 cm, and 225,000–270,000 hills ha ^−^ ^1^ (3–4 seedlings per hill). Basal fertilizer was applied as compound fertilizer, with water and fertilizer management consistent with the fresh edible pea-rice rotation model. Chemical pesticide use was minimized, with priority given to agricultural, physical, and biological control measures.

Crab culture: Juvenile crabs (100–200 individuals per kg) were stocked 15–20 days before rice transplanting at a density of 7,500–12,000 per ha (disinfected with saline before stocking). During the experiment, crabs were fed a combination of commercial formulated feed and locally available plant materials twice daily. Feeding rate was adjusted according to crab growth stage and field observations. Field water depth was maintained at 10–20 cm during most of the rice-growing period and adjusted according to rice and crab growth conditions, and water quality was monitored regularly (with periodic disinfection using quicklime). Crabs were harvested 10–15 days before rice harvest.

#### 2.4.3. Wheat-rice rotation.

Wheat cultivation: Sowing was conducted in early October (5 cm soil temperature stable at 15–18°C) with a seeding rate of 225–300 kg ha ^−^ ^1^, row spacing of 20–25 cm, and sowing depth of 3–5 cm. Basal fertilization combined compound fertilizer and organic fertilizer. Pre-winter management included gap filling, weeding, and overwintering irrigation; spring management involved topdressing at the green-up and jointing stages. Disease and pest control targeted key pests and diseases, and foliar nutrition was applied during grain filling. Harvesting was conducted in mid-June when over 90% of kernels matured.

Rice cultivation: Seedling raising was conducted in early April using factory-style methods. Transplanting was carried out from late June to early July with a wide-narrow row pattern (wide row 40 cm, narrow row 20 cm), plant spacing of 16–20 cm, and 225,000–270,000 hillsha ^−^ ^1^ (3–4 seedlings per hill). Basal fertilizer was applied as compound fertilizer, with water and fertilizer management and disease and pest control consistent with the fresh edible pea-rice rotation model. Harvesting was conducted in early to mid-October when over 95% of grains matured.

### 2.5. Statistical analysis

All experimental data were analyzed using SPSS 26.0 software (IBM Corp., Armonk, NY, USA). Prior to analysis, data were tested for normality and homogeneity of variance. One-way analysis of variance (ANOVA) was used to evaluate differences among cropping systems. When significant differences were detected, mean separation was performed using Tukey’s honestly significant difference (HSD) test at P < 0.05. Results are presented as mean ± standard deviation (SD).

### 2.6. Ethics, consent to participate, and consent to publish declarations

This study did not involve human participants, human data, human tissues, animals, or any procedures requiring ethical approval. Therefore, ethics approval, consent to participate, and consent to publish are not applicable.

## 3. Results

### 3.1. Economic benefit

#### 3.1.1. Fresh edible pea-rice rotation model.

The fresh edible pea-rice rotation system significantly enhanced economic performance compared with the rice monoculture control (Table 2). The combined production of fresh edible peas and rice increased land-use efficiency and overall economic return. Net income reached 9,966.75 CNY ha ^−^ ^1^, representing a 94.78% increase compared with rice monoculture. Although rice yield in the rotation system was slightly lower than that of the control treatment, the additional fresh pea production compensated for the yield difference and substantially improved total profitability.

**Table 2:** Yield and economic benefit of fresh edible pea-rice rotation.

Planting Pattern	Yield of Fresh Edible Peas (kg ha ^−^ ^1^, mean±SD)	Yield of Rice (kg ha ^−^ ^1^, mean±SD)	Total Input (CNY ha ^−^ ^1^)	Total Output Value (CNY ha ^−^ ^1^)	Net Income (CNY ha ^−^ ^1^, mean±SD)	Increase in Net Income Compared with Rice Monoculture (CNY ha ^−^ ^1^)	Increase Rate (%)	Statistical Significance
Fresh Edible Pea-Rice Rotation	26,213.1 ± 325.6a	10,155.15 ± 189.3a	43,500	53,466.75	9,966.75 ± 215.8a	4,849.95	94.78	a (P < 0.05)
Rice Monoculture (Control)		10,505.25 ± 201.5a	28,500	33,616.8	5,116.8 ± 156.2b			b (P < 0.05)

Note: Fresh edible pea purchase price: 0.4 CNY/kg; Rice purchase price: 1.6 CNY/kg.

#### 3.1.2. Rice-crab co-culture.

The rice-crab co-culture system produced the highest comprehensive economic benefit among the three cropping patterns (Table 3). Compared with rice monoculture, the co-culture system substantially increased net income due to the additional economic contribution from crab production. Net income ranged from 24,000–53,400 CNY ha ^−^ ^1^, which was markedly higher than that of conventional rice cultivation. Among the tested rice varieties, the Jinyuan U99–Chinese mitten crab combination achieved the highest overall economic return.

**Table 3:** Yield and economic benefit of rice-crab co-culture with different rice varieties.

Rice Variety	Rice Yield (kg ha ^−^ ^1^, mean±SD)	Crab Yield (kg ha ^−^ ^1^, mean±SD)	Total Input (CNY ha ^−^ ^1^)	Total Output Value (CNY ha ^−^ ^1^)	Net Income (CNY ha ^−^ ^1^, mean±SD)	Statistical Significance
Haiyu 7233	10,452.91 ± 245.7b	525 ± 32.1a	33,000-45,000	57,000-78,000	34,500 ± 2,150b	b (P < 0.05)
Jinyuan U99	11,022.66 ± 289.4a	540 ± 35.8a	33,000-45,000	60,000-98,400	40,200 ± 2,860a	a (P < 0.05)
Xiangjing 5	10,756.32 ± 267.5ab	510 ± 29.6a	33,000-45,000	58,500−88,200	34,350 ± 2,320b	b (P < 0.05)

Note: Rice purchase price: 1.4–1.76 CNY/kg; Crab purchase price: 80–120 CNY/kg.

#### 3.1.3. Wheat-rice rotation.

The wheat-rice rotation system improved annual grain productivity and economic return compared with conventional cropping systems (Table 4). Net income reached 17,729.1 CNY ha ^−^ ^1^, representing increases of 49.82% and 180.64% compared with the wheat-maize rotation and rice monoculture systems, respectively. The results indicate that integrating wheat and rice within an annual rotation system can effectively enhance land-use efficiency and stabilize grain production in moderately saline-alkali farmland.

**Table 4:** Yield and economic benefit of wheat-rice rotation.

Planting Pattern	Yield of Wheat (kg ha ^−^ ^1^, mean±SD)	Yield of Rice (kg ha ^−^ ^1^, mean±SD)	Yield of Corn (kg ha ^−^ ^1^, mean±SD)	Total Input (CNY ha ^−^ ^1^)	Total Output Value (CNY ha ^−^ ^1^)	Net Income (CNY ha ^−^ ^1^, mean±SD)	Statistical Significance
Wheat-Rice Rotation	9,004.5 ± 198.6a	8,254.2 ± 176.3a	–	40,500	58,229.1	17,729.1 ± 320.5a	a (P < 0.05)
Wheat-Corn Rotation (Control 1)	8,954.55 ± 210.3a	–	9,104.55 ± 235.7a	36,000	47,833.95	11,833.95 ± 285.3b	b (P < 0.05)
Rice Monoculture (Control 2)	–	9,204.6 ± 225.4a	–	28,500	34,817.4	6,317.4 ± 198.7c	c (P < 0.05)

Note: Wheat purchase price: 1.4 CNY/kg; Rice purchase price: 2.0 CNY/kg; Corn purchase price: 1.25 CNY/kg.

### 3.2. Ecological benefit

#### 3.2.1. Soil improvement.

All three cropping systems showed trends of improved soil physicochemical properties compared with the conventional monocropping systems (Table 5). Although no statistically significant differences (P > 0.05) were detected among treatments for any individual soil property indicator, all three systems exhibited positive changes relative to their respective controls: soil salinity decreased by 0.13%–0.20%, pH declined by 0.28–0.45 units, and soil organic matter increased by 0.20–0.30 percentage points after two consecutive growing seasons. The fresh edible pea-rice rotation promoted soil nitrogen accumulation through legume biological nitrogen fixation, while the rice-crab co-culture system showed numerically greater improvement in soil organic matter and structure-related indicators. These observed trends, while not reaching statistical significance within the two-year experimental period, suggest potential soil improvement benefits that may become more pronounced with longer-term implementation.

**Table 5:** Changes in soil properties after implementation of three planting patterns.

Planting Pattern	Soil Salinity Reduction (%)	Soil pH Reduction (Units)	Soil Organic Matter Increase (Percentage Points)	Soil Bulk Density Reduction (%)	Soil Porosity Increase (%)
Fresh Edible Pea-Rice Rotation	0.15 ± 0.03a	0.35 ± 0.08a	0.20 ± 0.05a	–	–
Rice-Crab Co-Culture	0.20 ± 0.04a	0.45 ± 0.09a	0.30 ± 0.06a	10.0 ± 1.5a	–
Wheat-Rice Rotation	0.13 ± 0.02a	0.28 ± 0.07a	0.23 ± 0.04a	–	12.5 ± 1.8a

#### 3.2.2. Reduction of environmental pollution.

Compared with conventional monocropping systems, all three cropping patterns reduced agrochemical inputs (Table 6). The rice-crab co-culture system exhibited the strongest pesticide reduction effect due to the combined use of biological and physical pest-control measures, with overall pesticide input decreasing by 35%–50% relative to controls (P < 0.05). Nitrogen and phosphorus loss reduction rates ranged from 17.5% to 22.5% across all treatments; however, these differences among treatments did not reach statistical significance (P > 0.05), suggesting that while the cropping patterns show potential for nutrient conservation benefits, longer-term observations with larger sample sizes may be needed to detect statistically robust treatment effects on nutrient losses.

**Table 6:** Reduction of agrochemical inputs and nutrient losses.

Planting Pattern	Pesticide Reduction Rate (%)(mean±SD)	Nitrogen Loss Reduction Rate (%)(mean±SD)	Phosphorus Loss Reduction Rate (%)(mean±SD)	Statistical Significance
Fresh Edible Pea-Rice Rotation	25.0 ± 3.2b	17.5 ± 2.1a	17.5 ± 2.0a	b (P < 0.05)
Rice-Crab Co-Culture	42.5 ± 4.5a	22.5 ± 2.8a	22.5 ± 2.5a	a (P < 0.05)
Wheat-Rice Rotation	25.0 ± 3.0b	20.0 ± 2.3a	20.0 ± 2.2a	b (P < 0.05)

#### 3.2.3. Enhancement of biodiversity.

The rice-crab co-culture system significantly enhanced biodiversity relative to the monoculture rice system (Table 7). The introduction of aquatic animals and reduced chemical pesticide application promoted the development of aquatic insect populations, plankton biomass, and soil microbial diversity. Compared with monoculture rice fields, aquatic insect species richness and microbial diversity increased by approximately 30%–40%.

**Table 7:** Biodiversity changes in rice-crab co-culture model.

Indicator	Monoculture Rice Field(mean±SD)	Rice-Crab Co-Culture Field(mean±SD)	Increase Rate (%)	Statistical Significance
Number of Aquatic Insect Species	9.0 ± 1.2b	13.0 ± 1.5a	33.3-50.0	a (P < 0.05)
Plankton Biomass (mg/L)	135.0 ± 15.3b	195.0 ± 18.6a	33.3-40.0	a (P < 0.05)
Soil Microbial Community Diversity (Shannon Index)	3.0 ± 0.2b	3.9 ± 0.3a	32.1-38.7	a (P < 0.05)

### 3.3. Social benefit (preliminary observations)

The following social benefit assessments are based on observational data and field records collected from the demonstration areas during the study period. These observations provide preliminary indications of potential social co-benefits associated with the evaluated cropping patterns; however, they were not derived from controlled experimental designs or formal socioeconomic surveys, and should therefore be interpreted as qualitative trends requiring further systematic investigation rather than statistically validated conclusions.

#### 3.3.1. Safeguarding food security.

The fresh pea-rice and wheat-rice rotation models increased total grain yield per ha by 15%−20% compared to monoculture. The wheat-rice rotation stabilized wheat production while maintaining rice output, and the rice-crab co-culture model preserved rice yield while adding aquatic products, enriching the agricultural product supply.

#### 3.3.2. Promoting employment and increasing farmers’ incomes.

Implementing the three models created additional labor demand: fresh pea-rice rotation required 75−120 additional labor days per ha, rice-crab co-culture needed 150−180 additional labor days per ha, and wheat-rice rotation added 45−75 additional labor days per ha. This provided 200−300 seasonal jobs per 66.67 ha of saline-alkali land, primarily for local farmers. The high net income of the models raised farmers’ annual income by 30%−50%.

#### 3.3.3. Advancing sustainable agriculture.

The models expanded cultivable area by improving moderately to strongly saline-alkali land (salt content 0.1%–0.5%, EC₁:₅ 2–4 dS m ^−^ ^1^), increasing effective agricultural land by 10%−15% in Tangshan. They promoted low-carbon agriculture [15] through reduced chemical inputs and straw recycling, lowering carbon emissions per ha by 15%−20%, and served as replicable models for other coastal regions.

## 4. Discussion

The observed improvements in soil properties can be attributed to several ecological mechanisms associated with different cropping systems [17]. In the pea-rice rotation system, biological nitrogen fixation by legumes enriches soil nitrogen pools and contributes to organic matter accumulation through root residues. In the rice-crab co-culture system, crab burrowing activities enhance soil aeration and reduce soil bulk density, while organic inputs from crab excreta contribute to nutrient cycling [18,19]. Long-term field studies have also reported that rice-crab coculture systems significantly reshape soil microbial communities and enhance soil ecological stability over time [20]. In the wheat-rice rotation system, alternating crops with different root architectures improves soil structure and promotes soil porosity. These mechanisms collectively facilitate salt leaching, improve soil structure, and enhance nutrient availability in coastal saline-alkali soils.

### 4.1. Novelty and localized adaptation of the Study

Although rice-crab co-culture and crop rotation systems have been previously reported, the novelty of this study lies not in proposing new planting models, but in providing a systematic and quantitative comparison of different planting patterns under the same coastal saline-alkali conditions. By integrating economic, soil physicochemical, and ecological indicators, this study elucidates how planting pattern selection shapes coordinated soil improvement and benefit formation processes under moderate to strongly saline conditions (EC₁:₅ 2–4 dS m ^−^ ^1^).

First, the results reveal distinct coupling mechanisms between soil salinity reduction, organic matter accumulation, and net economic return across different planting patterns. Rotation systems primarily promoted gradual soil improvement through biological nitrogen fixation and residue return, whereas the co-culture system enhanced soil structure and nutrient cycling through biological disturbance and organic inputs. These differences resulted in contrasting trajectories of soil improvement and income generation.

Second, this study provides a direct comparison between rotation-based systems and co-culture systems, demonstrating that co-culture achieves higher short-term comprehensive benefits, while rotation systems offer more stable soil improvement and lower input risks. This comparison clarifies the ecological-economic trade-offs among planting patterns in saline-alkali farmland, which have rarely been addressed in previous studies focusing on single systems.

Third, based on two consecutive growing seasons, this study quantifies the short-term response ranges of key soil indicators, including salinity reduction (0.13%−0.20%) and organic matter increase (0.20–0.30 percentage points), providing reference benchmarks for evaluating the effectiveness of planting pattern optimization in coastal saline-alkali lands. These quantified response ranges enhance the comparability and transferability of the results across regions with similar salinity conditions.

Similar rice-crab co-culture benefits have been documented in coastal Liaoning and Jiangsu provinces, where integrated rice-aquaculture systems simultaneously improved economic returns and soil ecological functions [17,21,22]. Previous studies have also demonstrated that rice-crab coculture systems can enhance crab growth performance, enzyme activity, and microbial diversity compared with conventional pond farming systems [23]. However, most previous studies focused on individual systems rather than direct comparisons among multiple cropping patterns. The present study extends previous research by quantitatively comparing rotation-based and co-culture systems under identical saline-alkali conditions, providing clearer evidence of their relative advantages and ecological-economic trade-offs.

### 4.2. Mechanisms of soil improvement and ecological benefits

The improved soil properties and ecological functions observed in this study can be attributed to distinct biological and physical mechanisms associated with each cropping system. In the pea-rice rotation system, biological nitrogen fixation by pea roots enriches soil nitrogen pools and increases organic carbon inputs through root and straw residues, thereby promoting soil fertility and mitigating salinity stress. In the rice-crab co-culture system, crab burrowing enhances soil aeration, reduces bulk density, and accelerates nutrient mineralization, while crab excreta serves as a natural organic amendment that increases soil organic matter and improves microbial activity. In the wheat-rice rotation system, alternating between upland and wetland crops improves soil aggregation and porosity, facilitating salt leaching and water infiltration. These mechanisms are consistent with previous reports that legume-involved rotations and rice-aquaculture co-cultures effectively improve soil structure and nutrient cycling in saline-alkali soils.

### 4.3. Long-term sustainability of soil improvement

Although significant reductions in soil salinity and improvements in soil quality were observed after two years, the long-term sustainability of these effects still needs further evaluation. Soil salinity in coastal saline-alkali land is affected by groundwater movement, climate conditions, and field management. Therefore, long-term responses may differ among cropping systems.

For the fresh edible pea–rice and wheat–rice rotation systems, continuous residue return and diverse root structures may gradually improve soil properties. Previous studies reported that crop rotation can increase soil organic carbon, improve microbial activity [24,25], and enhance soil quality over time. Legume-based rotations may also support long-term nutrient cycling through biological nitrogen fixation [26].

For the rice–crab co-culture system, ecological and economic benefits can appear more rapidly. Long-term studies showed that rice–crab systems can improve soil microbial communities and increase soil organic carbon accumulation [27,28]. However, excessive stocking density or high nutrient inputs may influence long-term system stability [29].

Overall, rotation systems may provide relatively stable and continuous soil improvement, while co-culture systems may generate faster ecological and economic returns. Long-term monitoring is still needed to determine whether these improvements can be maintained under different environmental conditions.

### 4.4. Comparison with previous studies

The present findings are generally consistent with previous studies reporting that rice-based rotation systems and integrated rice-aquaculture systems can simultaneously improve soil quality and economic return in saline-alkali regions.

Studies have shown that crop rotation systems, compared with continuous rice monoculture, can effectively enhance soil microbial biomass carbon (MBC), microbial biomass nitrogen (MBN), and the activities of various soil enzymes, such as dehydrogenase and urease. For example, in the maize–rice rotation system in the Terai region of India [30], the application of alternate tillage combined with vermicompost and chemical fertilizers significantly increased soil dehydrogenase and urease activities, while also enhancing total soil organic carbon content. In paddy soils of the Veneto region in Italy [31], rotation systems (soybean–rice–rice) significantly affected the activities of enzymes involved in carbon, nitrogen, phosphorus, and sulfur cycling, including β-glucosidase and arylsulfatase. Moreover, enzyme activities dynamically fluctuated with rice growth stages and soil moisture conditions, such as flooding and drainage. Long-term continuous cropping has negative effects on soil quality, whereas crop rotation can alleviate this trend. Studies conducted in oasis farmlands in China [32] found that long-term continuous cotton cultivation reduced soil enzyme activities and microbial diversity, while the introduction of crop rotation systems, such as wheat/sunflower rotation, significantly enhanced soil respiration rates and the activities of key soil enzymes. In Sri Lanka [33], the potato–fallow–vegetable–fallow rotation pattern exerted the most positive effects on soil hydraulic properties, thereby promoting crop growth.

Previous studies mainly focused on individual cropping systems, whereas the present study directly compared multiple representative cropping patterns under the same environmental conditions. This comparative approach provides clearer evidence regarding the ecological-economic trade-offs among different cropping systems in coastal saline-alkali farmland.

### 4.5. Limitations and future research

Although the present study demonstrated significant economic and ecological benefits of the three planting patterns over a two-year period, several uncertainties remain. First, the economic profitability of the systems may be influenced by market price fluctuations of agricultural products such as rice, peas, and crabs. Long-term monitoring under varying market conditions is necessary to evaluate the stability of economic returns. Second, the observed improvements in soil salinity, organic matter, and soil structure were derived from short-term field observations. Whether these improvements can be maintained or further enhanced over longer time scales requires continuous monitoring.

Future studies should therefore extend the experimental duration and integrate economic risk assessments, long-term soil monitoring, and multi-site trials to evaluate the sustainability and adaptability of these cropping systems in different coastal saline-alkali regions.

## 5. Conclusions

This study systematically evaluated the economic, ecological benefits and preliminary social observations of three planting patterns (fresh edible pea-rice rotation, rice-crab co-culture, wheat-rice rotation) in Tangshan's coastal saline-alkali lands through two-year field experiments. The results indicate that all three patterns demonstrate economic advantages over traditional monocropping in terms of net income. The fresh edible pea-rice rotation has significant economic advantages with a net income of 9,966.75 CNY ha ^−^ ^1^; the rice-crab co-culture achieves high comprehensive benefits with a net income of 24,000–53,400 CNY ha ^−^ ^1^ and remarkable ecological effects (30%−40% increase in biodiversity); the wheat-rice rotation stabilizes grain production and increases net income by 180.64% compared with rice monoculture. These models are suitable for different saline-alkali zones and provide replicable technical solutions for the efficient utilization of coastal saline-alkali land in the Bohai Bay region. Regarding soil quality improvements, the two-year study period revealed positive trends that did not reach statistical significance; longer-term monitoring is recommended to confirm whether these benefits accumulate over extended timeframes. This study addresses aspects of the key scientific questions proposed in the introduction, particularly regarding economic performance and ecological indicators (pesticide reduction), and provides quantitative reference for the optimization of cropping patterns in coastal saline-alkali lands with moderate to strongly saline conditions (EC₁:₅ 2–4 dS/m)

## Supporting information

S1 File This document contains Table S1–Table S6, including raw replicated data of crop yield, economic input and output, soil salinity improvement, agrochemical loss reduction and biodiversity under pea-rice rotation, rice-crab co-culture, wheat-rice rotation and monoculture control treatments. (DOCX)
