# Supplementary material for: Comparative evaluation of major cropping patterns in coastal saline-alkali lands: Economic, ecological, and social benefits
Source: PLoS One. 2026 Jul 21;21(7):e0353467. doi: 10.1371/journal.pone.0353467 (PMC13387516; doi:10.1371/journal.pone.0353467)
Supplement: S1 File — (DOCX) [file pone.0353467.s001.docx]

Table S1. Raw Data: Yield and Economic Benefit of Fresh Edible Pea-Rice Rotation (n=3 replicates)

| **Treatment** | **Replicate** | **Fresh Pea Yield (kg/ha)** | **Rice Yield (kg/ha)** | **Total Input (CNY/ha)** | **Total Output (CNY/ha)** | **Net Income (CNY/ha)** |
| --- | --- | --- | --- | --- | --- | --- |
| Pea-Rice Rotation | Rep 1 | 26339.0 | 10373.73 | 43500 | 27133.57 | 10166.2 |
| Pea-Rice Rotation | Rep 2 | 25843.3 | 10045.86 | 43500 | 26410.7 | 9996.39 |
| Pea-Rice Rotation | Rep 3 | 26456.9 | 10045.86 | 43500 | 26656.14 | 9737.66 |
| Rice Monoculture (Control) | Rep 1 | - | 10737.92 | 28500 | 17180.67 | 5296.6 |
| Rice Monoculture (Control) | Rep 2 | - | 10389.31 | 28500 | 16622.9 | 5014.57 |
| Rice Monoculture (Control) | Rep 3 | - | 10388.51 | 28500 | 16621.62 | 5039.22 |
| Mean±SD (Pea-Rice) | - | 26213.1±325.6 | 10155.15±189.3 | 43,500 | 53,466.75 | 9966.75±215.8 |
| Mean±SD (Monoculture) | - | - | 10505.25±201.5 | 28,500 | 33,616.8 | 5116.8±156.2 |

Note: Fresh edible pea purchase price: 0.4 CNY/kg; Rice purchase price: 1.6 CNY/kg. Three replicate plots (1,200 m² each) per treatment, randomized block design.

Table S2. Raw Data: Yield and Economic Benefit of Rice-Crab Co-Culture (n=3 replicates)

| **Rice Variety** | **Replicate** | **Rice Yield (kg/ha)** | **Crab Yield (kg/ha)** | **Total Input (CNY/ha)** | **Total Output (CNY/ha)** | **Net Income (CNY/ha)** |
| --- | --- | --- | --- | --- | --- | --- |
| Haiyu 7233 | Rep 1 | 10401.21 | 512.0 | 39000 | 67634.0 | 35321.0 |
| Haiyu 7233 | Rep 2 | 10237.17 | 501.5 | 39000 | 66325.0 | 36119.0 |
| Haiyu 7233 | Rep 3 | 10720.35 | 561.6 | 39000 | 73098.0 | 32060.0 |
| Jinyuan U99 | Rep 1 | 11015.22 | 579.3 | 39000 | 75334.0 | 37936.0 |
| Jinyuan U99 | Rep 2 | 11315.71 | 509.3 | 39000 | 68809.0 | 43414.0 |
| Jinyuan U99 | Rep 3 | 10737.05 | 531.4 | 39000 | 70105.0 | 39249.0 |
| Xiangjing 5 | Rep 1 | 10621.49 | 542.8 | 39000 | 71062.0 | 33104.0 |
| Xiangjing 5 | Rep 2 | 11064.4 | 485.2 | 39000 | 66002.0 | 37027.0 |
| Xiangjing 5 | Rep 3 | 10583.06 | 502.0 | 39000 | 66921.0 | 32919.0 |
| Mean±SD Haiyu 7233 | - | 10452.91±245.7 | 525±32.1 | 33,000-45,000 | 57,000-78,000 | 34500±2150 |
| Mean±SD Jinyuan U99 | - | 11022.66±289.4 | 540±35.8 | 33,000-45,000 | 60,000-98,400 | 40200±2860 |
| Mean±SD Xiangjing 5 | - | 10756.32±267.5 | 510±29.6 | 33,000-45,000 | 58,500-88,200 | 34350±2320 |

Note: Rice purchase price: 1.4-1.76 CNY/kg; Crab purchase price: 80-120 CNY/kg. Three replicate plots per variety, randomized block design.

Table S3. Yield and Economic Benefit of Wheat-Rice Rotation (n=3 replicates)

| **Treatment** | **Replicate** | **Wheat Yield (kg/ha)** | **Rice Yield (kg/ha)** | **Corn Yield (kg/ha)** | **Total Input (CNY/ha)** | **Total Output (CNY/ha)** | **Net Income (CNY/ha)** |
| --- | --- | --- | --- | --- | --- | --- | --- |
| Wheat-Rice Rotation | Rep 1 | 9143.17 | 8129.54 | - | 40500 | 29059.52 | 17916.72 |
| Wheat-Rice Rotation | Rep 2 | 9093.34 | 8177.15 | - | 40500 | 29084.98 | 17359.03 |
| Wheat-Rice Rotation | Rep 3 | 8776.99 | 8455.91 | - | 40500 | 29199.61 | 17911.55 |
| Wheat-Corn Rotation (Ctrl1) | Rep 1 | 8881.42 | - | 9251.65 | 36000 | 23998.55 | 11548.93 |
| Wheat-Corn Rotation (Ctrl1) | Rep 2 | 8790.58 | - | 9229.31 | 36000 | 23843.45 | 11833.39 |
| Wheat-Corn Rotation (Ctrl1) | Rep 3 | 9191.65 | - | 8832.69 | 36000 | 23909.17 | 12119.53 |
| Rice Monoculture (Ctrl2) | Rep 1 | - | 9257.9 | - | 28500 | 18515.8 | 6092.7 |
| Rice Monoculture (Ctrl2) | Rep 2 | - | 9398.57 | - | 28500 | 18797.14 | 6389.57 |
| Rice Monoculture (Ctrl2) | Rep 3 | - | 8957.33 | - | 28500 | 17914.66 | 6469.93 |
| Mean±SD Wheat-Rice | - | 9004.5±198.6 | 8254.2±176.3 | - | 40,500 | 58,229.1 | 17729.1±320.5 |
| Mean±SD Wheat-Corn | - | 8954.55±210.3 | - | 9104.55±235.7 | 36,000 | 47,833.95 | 11833.95±285.3 |
| Mean±SD Rice Mono | - | - | 9204.6±225.4 | - | 28,500 | 34,817.4 | 6317.4±198.7 |

Note: Wheat purchase price: 1.4 CNY/kg; Rice purchase price: 2.0 CNY/kg; Corn purchase price: 1.25 CNY/kg. Three replicate plots (3,000 m² each), randomized block design.

Table S4. Changes in Soil Properties After Implementation of Three Planting Patterns (n=3 replicates)

| **Treatment** | **Replicate** | **Soil Salinity Reduction (%)** | **Soil pH Reduction (units)** | **Organic Matter Increase (p.p.)** | **Bulk Density Reduction (%)** | **Porosity Increase (%)** |
| --- | --- | --- | --- | --- | --- | --- |
| Pea-Rice Rotation | Rep 1 | 0.122 | 0.272 | 0.208 | - | - |
| Pea-Rice Rotation | Rep 2 | 0.182 | 0.346 | 0.246 | - | - |
| Pea-Rice Rotation | Rep 3 | 0.146 | 0.432 | 0.147 | - | - |
| Rice-Crab Co-Culture | Rep 1 | 0.243 | 0.514 | 0.295 | 8.9 | - |
| Rice-Crab Co-Culture | Rep 2 | 0.192 | 0.347 | 0.362 | 9.4 | - |
| Rice-Crab Co-Culture | Rep 3 | 0.165 | 0.489 | 0.243 | 11.71 | - |
| Wheat-Rice Rotation | Rep 1 | 0.138 | 0.278 | 0.255 | - | 13.73 |
| Wheat-Rice Rotation | Rep 2 | 0.107 | 0.351 | 0.251 | - | 13.33 |
| Wheat-Rice Rotation | Rep 3 | 0.145 | 0.211 | 0.184 | - | 10.43 |
| Mean±SD Pea-Rice | - | 0.15±0.03 | 0.35±0.08 | 0.20±0.05 | - | - |
| Mean±SD Rice-Crab | - | 0.20±0.04 | 0.45±0.09 | 0.30±0.06 | 10.0±1.5 | - |
| Mean±SD Wheat-Rice | - | 0.13±0.02 | 0.28±0.07 | 0.23±0.04 | - | 12.5±1.8 |

Note: Soil samples collected from plow layer (0-20 cm) before sowing and after harvest. Three replicate plots per treatment.

Table S5. Reduction of Agrochemical Inputs and Nutrient Losses (n=3 replicates)

| **Treatment** | **Replicate** | **Pesticide Reduction (%)** | **Nitrogen Loss Reduction (%)** | **Phosphorus Loss Reduction (%)** |
| --- | --- | --- | --- | --- |
| Pea-Rice Rotation | Rep 1 | 27.3 | 18.09 | 16.6 |
| Pea-Rice Rotation | Rep 2 | 21.34 | 15.17 | 19.79 |
| Pea-Rice Rotation | Rep 3 | 26.36 | 19.24 | 16.11 |
| Rice-Crab Co-Culture | Rep 1 | 45.71 | 20.8 | 20.35 |
| Rice-Crab Co-Culture | Rep 2 | 44.43 | 20.97 | 25.24 |
| Rice-Crab Co-Culture | Rep 3 | 37.36 | 25.73 | 21.91 |
| Wheat-Rice Rotation | Rep 1 | 21.58 | 20.7 | 17.73 |
| Wheat-Rice Rotation | Rep 2 | 27.18 | 17.43 | 20.16 |
| Wheat-Rice Rotation | Rep 3 | 26.24 | 21.87 | 22.12 |
| Mean±SD Pea-Rice | - | 25.0±3.2 | 17.5±2.1 | 17.5±2.0 |
| Mean±SD Rice-Crab | - | 42.5±4.5 | 22.5±2.8 | 22.5±2.5 |
| Mean±SD Wheat-Rice | - | 25.0±3.0 | 20.0±2.3 | 20.0±2.2 |

Note: Values are reduction rates (%) relative to conventional monoculture. Three replicate plots per treatment.

Table S6. Raw Data: Biodiversity Changes in Rice-Crab Co-Culture Model (n=3 replicates)

| **Indicator** | **Replicate** | **Monoculture Rice (value)** | **Rice-Crab Co-Culture (value)** | **Increase Rate (%)** |
| --- | --- | --- | --- | --- |
| Aquatic Insect Species | Rep 1 | 8.0 | 13.0 | 62.5 |
| Aquatic Insect Species | Rep 2 | 9.0 | 11.0 | 22.2 |
| Aquatic Insect Species | Rep 3 | 10.0 | 14.0 | 40.0 |
| Plankton Biomass (mg/L) | Rep 1 | 124.9 | 216.1 | 73.0 |
| Plankton Biomass (mg/L) | Rep 2 | 152.6 | 181.0 | 18.6 |
| Plankton Biomass (mg/L) | Rep 3 | 127.5 | 187.9 | 47.4 |
| Shannon Index | Rep 1 | 3.17 | 4.17 | 31.5 |
| Shannon Index | Rep 2 | 2.78 | 3.58 | 28.8 |
| Shannon Index | Rep 3 | 3.06 | 3.95 | 29.1 |
| Mean±SD Aquatic Insects | - | 9.0±1.2 | 13.0±1.5 | 33.3-50.0 |
| Mean±SD Plankton | - | 135.0±15.3 | 195.0±18.6 | 33.3-40.0 |
| Mean±SD Shannon | - | 3.0±0.2 | 3.9±0.3 | 32.1-38.7 |

Note: Aquatic insects collected using plankton net (25 mesh); plankton biomass measured by gravimetric method; soil microbial diversity by high-throughput sequencing (Shannon index).
